# Assessment of the Tribological Performance of Electrospun Lignin Nanofibrous Web-Thickened Bio-Based Greases in a Nanotribometer

**DOI:** 10.3390/nano13212852

**Published:** 2023-10-27

**Authors:** María Borrego, Erik Kuhn, José E. Martín-Alfonso, José M. Franco

**Affiliations:** 1 Chemical Product and Process Technology Research Center (Pro2TecS), Department Chemical Engineering and Materials Science, Escuela Técnica Superior de Ingeniería (ETSI), Campus de “El Carmen”, University of Huelva, 21071 Huelva, Spain; maria.borrego@diq.uhu.es (M.B.); jose.martin@diq.uhu.es (J.E.M.-A.); 2 Laboratory of Machine Elements and Tribology, Department of Mechanical Engineering and Production, Faculty of Engineering Technology and Computer Science, Hamburg University of Applied Sciences (HAW-Hamburg), Berliner Tor 21, 20099 Hamburg, Germany; erik.kuhn@haw-hamburg.de

**Keywords:** bio-based lubricating greases, electrospinning, friction, lignin, nanofiber, wear

## Abstract

The tribological performance of novel bio-based lubricating greases thickened with electrospun lignin nanostructures was investigated in a nanotribometer using a steel–steel ball-on-disc configuration. The impact of electrospun nanofibrous network morphology on friction and wear is explored in this work. Different lignin nanostructures were obtained with electrospinning using ethylcellulose or PVP as co-spinning polymers and subsequently used as thickeners in castor oil at concentrations of 10–30% wt. Friction and wear generally increased with thickener concentration. However, friction and wear decreased when using homogeneous bead-free nanofiber mats (with higher fiber diameter and lower porosity) rather than nanostructures dominated by the presence of particles or beaded fibers, which was favored by reducing the lignin:co-spinning polymer ratio.

## 1. Introduction

The main purpose of lubrication is the reduction in energy losses caused by friction in machine elements and wear minimization, thus prolonging the reliability and service life of mechanical components and equipment [1]. In addition, in the case of lubricating greases, the rheological characteristics imparted by the thickener microstructure make this particular lubricant especially appropriate in some applications where decreasing leakage and the frequency of lubrication are desired, especially in hermetic or hard-to-reach contacts, or when vibrations and fluctuations with temperature and loads must be minimized [2]. Moreover, lubricating greases act as effective seals against contaminants, such as particles and water [3].

As extensively reported by Lugt and co-workers [4,5,6,7], the lubrication mechanism of greases is extremely complex and affected by many factors such as the inherent complex rheological properties of greases, geometry of the lubricated contacts and components and the different coexisting shear and extension rates internally achieved. In most cases, grease lubrication involves the mixed or elastohydrodynamic (EHL) lubrication regimes, where the film is not only determined by the lubricant properties. Although several aspects are still under discussion, it is generally accepted that the grease lubrication mechanism is mainly governed by the replenishment of the lubricant into the contact and the oil bleeding ability, i.e., the capacity of the grease microstructure to release oil [8,9,10]. In this sense, the grease microstructure would act as a sponge that replenishes the lubricated contact with the bled oil once submitted to an external load [8,9,11]. Different models considering the morphology of grease microstructure and/or thickener particle geometry have been reported [9,12]. However, it is also generally assumed that the thickener exerts a significant impact in grease lubrication, not only acting as a reservoir for the oil but penetrating into the contact and affecting the film thickness, either thickening the bled oil or as isolated particles, eventually influencing friction and wear [1,13,14,15,16,17]. Both effects, the oil bleeding ability and thickener penetration, are largely determined by the grease microstructure imparted by the thickener, which may be also influenced by its mechanical degradation [18,19,20]. Therefore, the correct elucidation of grease microstructure, as well as the effect of shearing on this, is essential to understanding the lubrication mechanisms [17,21,22].

On the other hand, as a consequence of environmental awareness, there is a widespread concern in the lubricant industry to replace petroleum-based components with others, biodegradable or obtained from natural resources. Thus, the number of scientific and technical research related to the development of biolubricants from natural resources has increased accordingly over the last two decades, as can be deduced from the vast number of review papers recently published on this topic [23,24,25,26,27]. In the case of lubricating greases, since the main component is the base oil and preserving the functionality associated with thickener microstructure is essential for proper lubrication, the main initiatives to achieve biodegradable formulations have been focused on the replacement of traditional mineral oils with vegetable-derived oils or glycerol esters, while maintaining metallic thickeners in the formulation [23,28,29]. However, given that the thickener content in greases can vary between 3% and 40% by weight, and that the thickeners with the best technical performance and most widely used in industry are metal soaps and polyureas, especially lithium soaps, there is a clear need to develop new renewable thickening agents that may provide adequate technical performance. In addition, the importance of lithium in other more demanding and priority applications like batteries and the supply constraints associated with this scarce resource must be taken into account. Therefore, the development of bio-based thickeners obtained from natural and renewable polymers, making them technologically efficient, represents an environmentally friendly alternative to metal soaps and synthetic polymers derived from the petrochemical industry (polyurea, polyurethanes, polypropylene, etc.) that are not biodegradable and/or require highly toxic or polluting production processes. Unfortunately, the oil structuring ability of biopolymers such as cellulose and chitosan derivatives or lignocellulosic components is very limited due to their polar chemical nature. Only very few biopolymers, like ethylcellulose, are able to gel oils directly by the formation of supramolecular structures [30] but still exhibit serious drawbacks as effective thickeners in lubricant formulations [31].

In previous investigations, different strategies involving chemical modifications of biopolymers to reduce their polarity and enhance oil affinity [32] or to induce oil structuration via chemical crosslinking with isocyanate or epoxide reactive groups [33,34] have been proposed. Three-dimensional structural networks that perfectly mimic the grease microstructure provided by lithium soaps, were for instance achieved with lignocellulose modified with diisocyanates [33]. Nevertheless, these chemical modifications usually require chemicals, solvents and/or catalysts that make the synthesis of biopolymeric thickeners and the subsequent oil structuring process relatively complex and not entirely environmentally friendly, despite the fact the final product is. Very recently, we demonstrated that nanostructures generated with electrospinning from non-modified biopolymers can be proposed as effective oil structuring agents [35,36]. The high porosity, small size and high surface/volume ratio of nanofibrous webs are able to induce the formation of three-dimensional networks with a superior capacity to promote physical interactions between the oil and the nanofibers stabilizing the colloidal system through the formation of a percolation network.

Lignin is an attractive biopolymer due to the fact that is considered a waste or low-value by-product resulting from different biorefinery processes, which has been previously employed to produce oil thickening agents via chemical crosslinking [37]. However, producing lignin nanofibers using electrospinning is particularly challenging as a consequence of its chemical structure and relatively low molecular weight [38], and often requires the use of a co-spinning polymer [35,39]. However, non-uniform nanostructures typically obtained with lignins, consisting of particles or globules distributed along the filaments, which have come to be called BOAS (beads-on-a-string) [40,41] or weakly mixed particle–fiber nanostructures might also be appropriate for lubricating purposes, in terms of an easy oil release, i.e., oil bleeding [36]. The aim of this work is to investigate the tribological performance of novel bio-based lubricating greases, thickened with different electrospun lignin nanostructures, in a ball-on-disc nanotribometer by addressing the impact of the electrospun network morphology on friction and wear. A nanotribometer was selected because of its sensitivity in measuring weak effects due to small variations in thickener nanostructures.

## 2. Materials and Methods

### 2.1. Materials

A softwood low-sulphonate lignin (LSL) from Merck Sigma-Aldrich (St. Louis, MO, USA) (M_w_: ~10,000 g/mol) was used as the main polymer to produce structural networks using electrospinning and subsequently thicken castor oil (viscosity: 0.55 Pa·s, density: 0.958 g/mL, at 25 °C) supplied by Guinama (Valencia, Spain). High molecular weight ethyl cellulose (EC, M_w_ = 8.2 × 10^4^ g/mol) and polyvinylpyrrolidone (PVP, M_w_: ~ 3.6 × 10^5^ g/mol) purchased from Merck Sigma-Aldrich, were used as co-spinning polymers. Tetrahydrofuran (THF, purity 99.0%), dimethylacetamide (DMAc, 99.8% purity) and dimethylformamide (DMF, 99.8% purity), also supplied by Sigma-Aldrich (St. Louis, MO, USA), were used as solvents.

### 2.2. Electrospinning of Lignin Solutions

Solutions of LSL/EC in a DMAc:THF (1:1 *w*/*w*) binary solvent system were prepared at 10 and 15% wt. total concentration by modifying the LSL:EC ratio (50:50, 70:30 and 90:10), under agitation at 40 °C for 24 h, using a magnetic stirrer, at 500 rpm, on a hot plate. LSL/PVP solutions were similarly prepared in DMF at 10% wt. total concentration at the same weight ratios (50:50, 70:30 and 90:10). These solutions were used as feed for electrospinning in a device constructed using a high-voltage power supply (Spellman High Voltage Electronics Corporation, Hauppauge, NY, USA), a syringe with a blunt metal needle, a syringe pump (KD Scientific Pump Company, Holliston, MA, USA) and a grounded aluminum foil collector. The polymeric solution was fed through the needle tip with a syringe pump at a flow rate of 0.8–2 mL/h (tip diameter ≈ 0.6 mm). Nanostructures were produced at a tip-to-target distance of 12 cm and an applied voltage of 12–20 kV. All the electrospinning experiments were carried out at 23 °C (±2 °C) with a relative humidity of 45% (±5%). The relative humidity was roughly controlled by inserting a dehumidifier containing a saturated Mg(NO_3_)_2_ solution in the electrospinning chamber.

The morphology of the resulting electrospun fiber webs was examined using scanning electron microscopy (FlexSEM 1000 II, Hitachi, Tokyo, Japan) after sputtering the samples with gold under vacuum. SEM experiments were carried out at accelerating voltages of 10–20 kV. The FIJI ImageJ analysis software (version 1.52p, 2019) was used to analyze the SEM pictures. Random observations were carried out at the same magnification to determine the average diameter of particles and fibers and the porosity of the electrospun nanostructure by adjusting the contrast of the micrographs.

### 2.3. Preparation of Bio-Based Greases with Electrospun Lignin Nanostructures

Electrospun lignin nanostructures obtained using electrospinning were used to thicken castor oil by simply dispersing the solid collected from the electrospinning device under gentle mechanical agitation (60 rpm, during 60 min) using an anchor impeller geometry at room temperature (~23 °C). Electrospun lignin nanostructures were incorporated at three different concentrations (10, 20 and 30%). These dispersions were stored at room temperature and rest conditions for at least 24 h to assess physical stability, i.e., no phase separation, previously to carry out the rheological and tribological characterization. Only the dispersion of nanostructures formed with 90:10 LSL/co-spinning polymer ratios obtained from solutions at 10% wt. evinced a certain degree of oil separation after several days. The oil separation ability was studied using centrifugation. Approximately 0.7 g of each selected sample was centrifuged in a Sorvall ST8 (Thermo Fisher Scientific, Waltham, MA, USA) centrifuge at 16,000 rpm (equivalent to 24,328× *g*) for 30 min, and the amount of separated oil was subsequently weighed.

### 2.4. Tribological Characterization

The tribological performance of the model greases thickened with electrospun lignin nanostructures was investigated in a ball-on-disc CSM nanotribometer (Peseux, Switzerland). The set-up basically consists of a static ball, which is fixed to a cantilever to avoid rolling, and a movable disc, which is stressed by applying a normal force. A fiber-optic sensor measures the deflection of the cantilever in the radial direction to determine the frictional force. A more detailed description of the equipment and experimental setup can be found elsewhere [42]. All tests were carried out in the rotational mode under pure sliding motion (sliding speed: 40 mm·s^−1^) in a steel–steel (115CrV3, hardness 22.72 HRC) ball-on-disc configuration, using a 1 mm diameter ball. Three normal loads (400, 700 and 900 mN, corresponding to 1.60, 1.93 and 2.10 GPa Hertzian pressures, respectively) were applied during a test duration of 20 min. The examined greases were applied to the disc surface prior to the start of each test without manual relubrication during the test. Each test was replicated ten times on the same track at room temperature (21 ± 1 °C). The dimensions of the wear grooves on the steel plates created after the ten repetitions of the test were quantified using the white light interferometry technique using a Zygo Nexview apparatus (Middlefield, OH, USA) and Zygo Mx software (version 7.1.0.4, 2017). The wear volume values reported hereunder correspond to a groove length of 100 μm. Obviously, wear also occurs to some extent on the balls. However, wear marks in the 1 mm balls were small, and the results did not discriminate between the different greases. Therefore, wear data of the ball surface have not been considered.

### 2.5. Rheological Characterization

Model greases were rheologically characterized using a MARS controlled-stress rheometer (ThermoHakee, Uhingen, Germany), using serrated parallel plate (20 and 35 mm; 1 mm gap) geometries to avoid possible slip effects. Viscous flow tests were performed, at 25 °C, by applying stepped shear rate ramps from 0.02 to 300 s^−1^. At least two replicates of each test were performed.

## 3. Results and Discussion

### 3.1. Morphology of Electrospun Lignin Nanostructures

**Figure 1:**
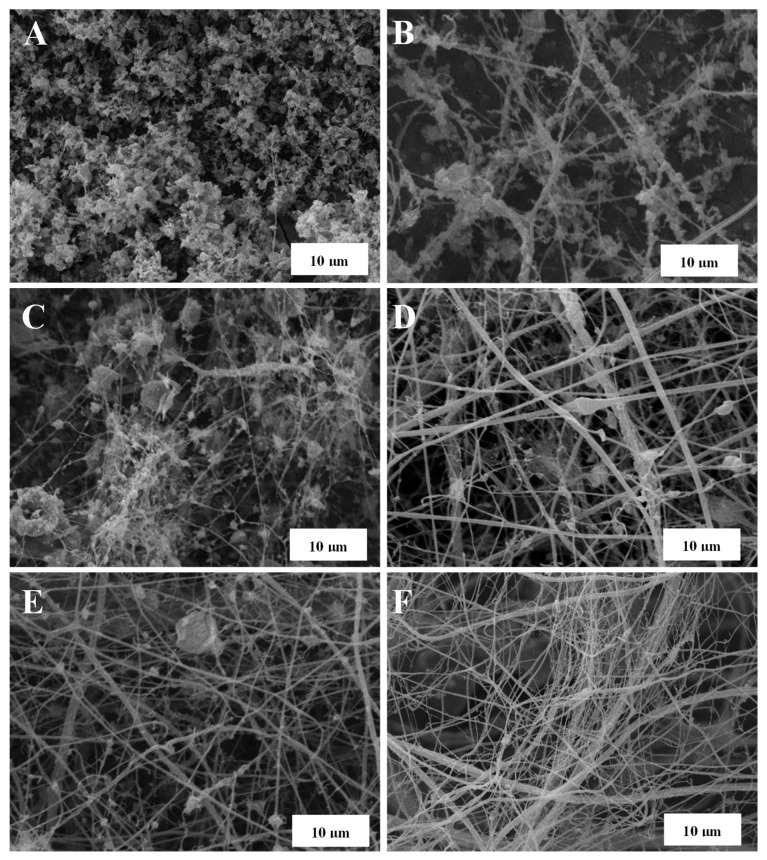
SEM images of electrospun LSL/EC nanostructures obtained with different LSL:EC ratios and concentrations of the feeding solution: (**A**) 90:10, 10% solution concentration, (**B**) 90:10, 15% solution concentration, (**C**) 70:30, 10% solution concentration, (**D**) 70:30, 15% solution concentration, (**E**) 50:50, 10% solution concentration and (**F**) 50:50, 15% solution concentration.

**Figure 2:**
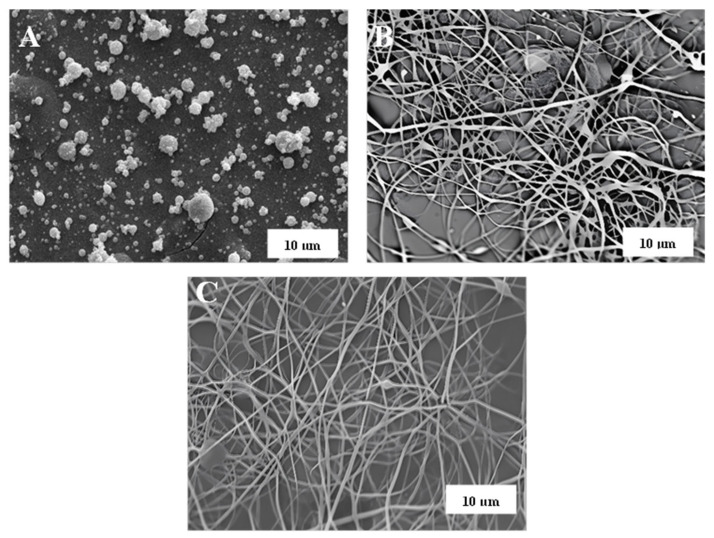
SEM Images of electrospun LSL/PVP nanostructures obtained with different LSL:PVP ratios: (**A**) 90:10, (**B**) 70:30 and (**C**) 50:50 (feeding solution concentration: 10% wt.).

The morphology of LSL/EC nanostructures subsequently used to thicken castor oil largely depends on the concentration of the feeding solution and the LSL:EC ratio, as illustrated in Figure 1. As can be seen, an evolution from agglomerated submicrometric electrosprayed particles to the typical BOAS (beads-on-a-string) structure dominated by nanofibers was found when increasing the solution concentration or decreasing the LSL:EC ratio. The lower the LSL:EC ratio, the lower the number of beads that appear in the nanofibrous web. Finally, when feeding the electrospinning chamber with a solution having high concentration and a low LSL:EC ratio, i.e., 15% wt. and 50:50 LSL:EC ratio, bead-free nanofiber webs were obtained (see Figure 1F). Different lignin nanostructures were also obtained using PVP as a co-spinning polymer and modifying the LSL:PVP weight ratio in the feeding solution. As can be observed in Figure 2, a similar evolution in the morphology was detected when increasing the amount of PVP in the nanostructure, although generally, more homogeneous nanofiber mats and a lower amount of particle beads were detected when replacing EC with PVP as the co-spinning polymer (compare, for instance, Figure 2B,C with Figure 1C,E, respectively, for the same feeding solution concentration and electrospinning conditions). Further details on the relationship between the physicochemical properties of the lignin solutions and the morphology of the resulting nanostructures can be found elsewhere [35].

### 3.2. Rheological Behavior of Electrospun Nanostructure-Thickened Lubricating Greases

The electrospun lignin nanostructures described above were used to thicken castor oil, aiming to impart gel-like characteristics and obtain model bio-based greases. Regardless of the thickener concentration and the LSL:co-spinning polymer ratio, all greases showed a similar shear-thinning response with a tendency to reach a constant high shear-rate limiting viscosity. Experimental viscous flow curves were satisfactorily fitted (R^2^ < 0.995) to the Sisko model:

(1) $$\eta =K{\dot{\gamma }}^{n-1}+\eta_{\infty }$$

where *η* is the non-Newtonian viscosity, $\dot{\gamma }$ is the shear rate, *K* and *n* are the consistency and flow indexes, respectively, and *η*_∞_ is the high shear-rate limiting viscosity. Table S1 in the Supplementary Materials collects the values of Sisko parameters obtained from the fits. In general, *K* and *η*_∞_ values clearly increased with the thickener concentration, i.e., the content of electrospun nanostructures, and decreased with the LSL:co-spinning polymer ratio, whereas *n* generally decreases with increasing the thickener concentration or the co-spinning polymer proportion. Figures S1 and S2 in the Supplementary Materials illustrate the effect of both these compositional variables on the viscous flow curves of the bio-greases studied. According to the morphological characteristics of the electrospun nanostructures, the more homogeneous and well-developed nanofiber mats and the lower amount of particle beads, the higher the viscosity of the colloidal dispersion, reflected in higher *K* and *η*_∞_ values.

### 3.3. Influence of Concentration on the Tribological Properties of Electrospun Nanostructure-Thickened Lubricating Greases

**Figure 3:**
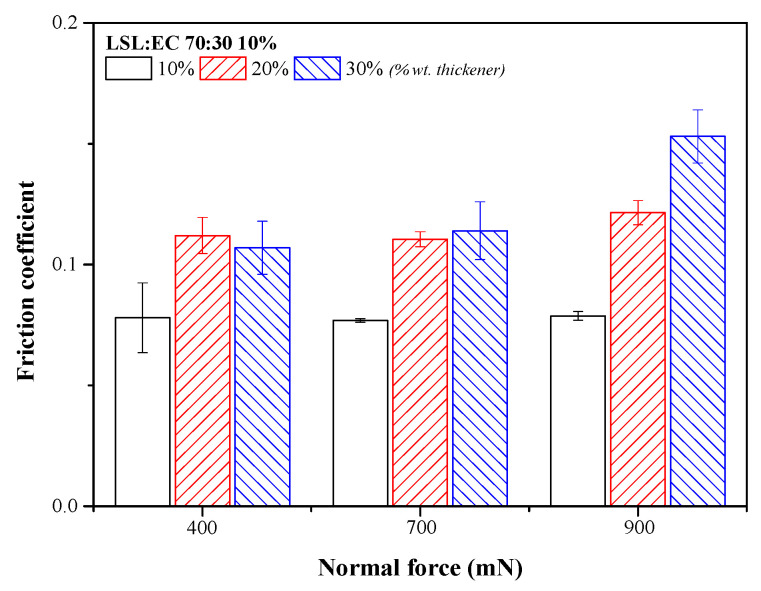
Friction coefficient values obtained with lubricating greases thickened with the 70:30 LSL/EC electrospun nanostructure at different concentrations (10%, 20% and 30% wt.).

Electrospun lignin nanostructures were dispersed in castor oil at three different concentrations (10, 20 and 30% wt.), and the resulting greases were tested in a steel–steel ball-on-disc nanotribometer. Figure 3 shows the values of the friction coefficient, measured at different normal forces, using greases thickened with the 70:30 LSL:EC nanostructure as the lubricant. In general, it is worth noting a significant increase in the friction coefficient with the nanostructure concentration, which is more evident at high normal force. Moreover, the friction coefficient is not significantly affected by the normal force when using a low thickener concentration or slightly increases with this at higher concentrations, which confirms that the experiments were conducted under mixed lubrication conditions. Furthermore, the friction coefficient values obtained when using these novel greases as lubricants are significantly higher than those obtained with the nanofiber-free base oil (see the values in Table S2). This is a well-known result in the mixed lubrication regime since friction is caused by both the contact between the surface asperities and by the contribution of the lubricant, which, in the case of greases, includes the internal friction of the microstructure.

**Figure 4:**
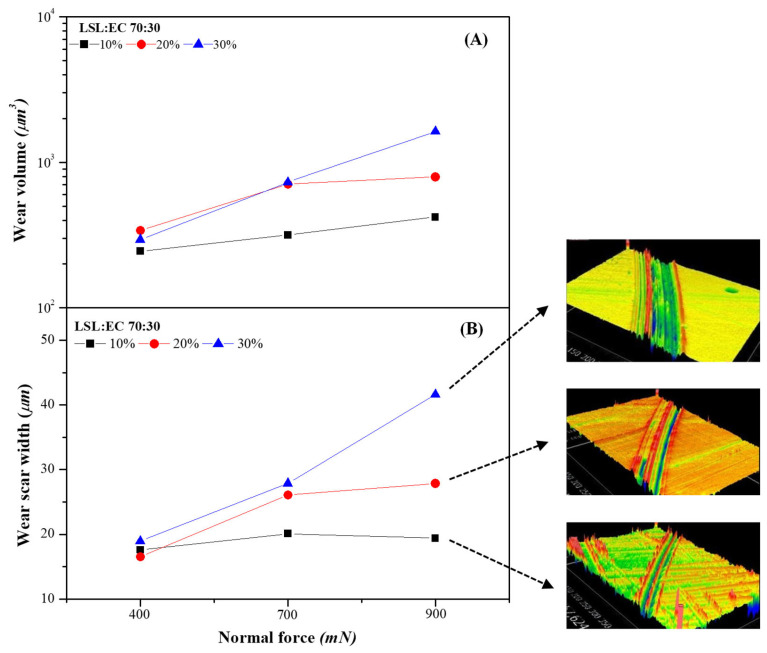
Evolution of the average scar wear volume (**A**) and wear width (**B**) with the normal force applied when using lubricating greases thickened with the 70:30 LSL/EC electrospun nanostructure (10% wt. feeding solution) at different concentrations (10%, 20% and 30% wt.).

Aiming to explain the influence of lignin nanostructure concentration on the friction coefficient, two phenomena must be taken into account. As extensively discussed by Gonçalves et al. [13,43,44], the friction coefficient values typically increase with a decrease in the entrainment speed, which is related to the grease replenishment into the contact. The contact replenishment capacity is probably reduced by increasing the thickener concentration as a result of a significantly increased grease consistency (see rheological parameters in Table S1 in the Supplementary Materials). The oil bleeding ability is generally dampened by increasing the thickener concentration since the three-dimensional network entraps the oil more effectively [12,13]. For selected samples, Table S3 in the Supplementary Materials shows that after centrifugation oil separation is favored by decreasing thickener concentration. On the other hand, the penetration of the thickener into the contact increases the film thickness and must reduce the friction coefficient [14,44], as it must occur when reducing the normal force applied (see Figure 3). However, the increase in nanostructure concentration does not seem to favor the thickener penetration or, if this penetration occurs, it does not exert a friction reduction. It must be taken into account that lignin, especially particles or beads, is somewhat abrasive and may negatively affect the mating surface, contributing to increased friction and wear, similar to what happens with debris particles or contaminants in the tribological contact [45]. The effects of the normal force under mixed conditions and the possible penetration of lignin nanofibers into the contact were also elucidated by analyzing the wear tracks on the plates measured using interferometry. Figure 4 shows the effect of thickener concentration on the evolution of both the wear groove volume and the average wear width as a function of the applied normal force. As can be seen, wear is not noticeably influenced by lignin concentration at the lowest normal force (400 mN), but significantly increases with this for higher normal forces, when the lubrication mechanism seems to be mainly governed by the oil bleeding ability. A similar thickener concentration influence on friction and wear was previously reported when using epoxidized lignin [46] or nanosized montmorillonite [47] as thickeners in castor oil or cellulose acetate butyrate in acetyl tributyl citrate and triethyl citrate media, respectively [48,49].

### 3.4. Influence of the LSL:EC Ratio on the Tribological Properties of Electrospun Nanostructure-Thickened Lubricating Greases

**Figure 5:**
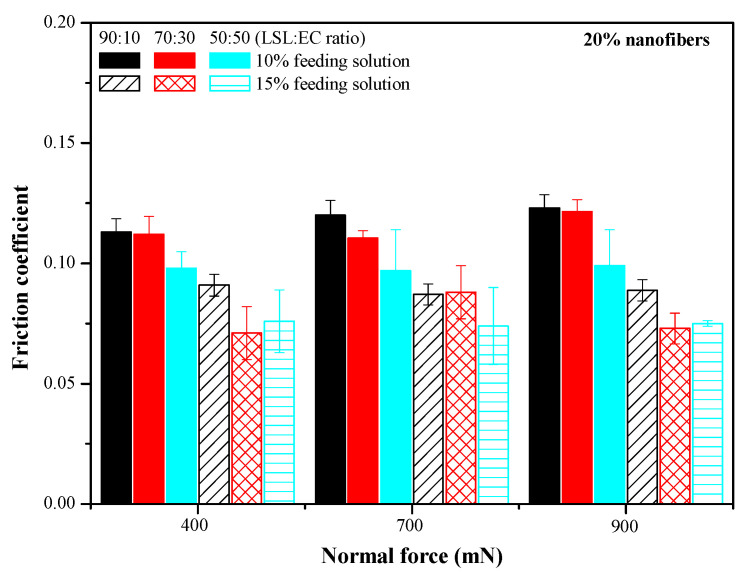
Friction coefficient values obtained with lubricating greases thickened with LSL/EC electrospun nanostructures differing in the LSL:EC weight ratio (thickener concentration 20% wt.).

**Figure 6:**
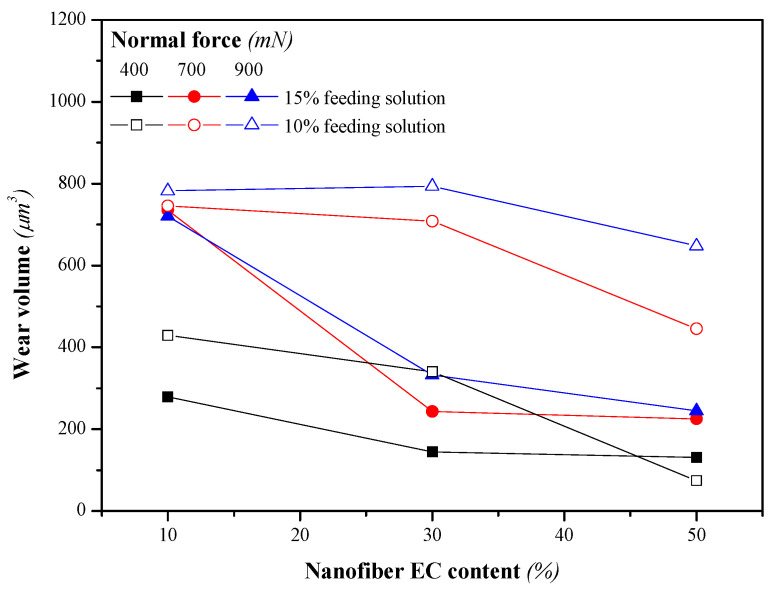
Wear volume produced when using lubricating greases thickened with LSL/EC electrospun nanostructures differing in the LSL:EC weight ratio (thickener concentration: 20% wt.).

Figure 5 shows the values of the friction coefficient obtained for the three normal forces applied when using bio-based greases thickened with LSL/EC nanostructures, at 20% wt., as a function of the LSL:EC ratio (50:50, 70:30 and 90:10) and the initial feeding solution concentration (10 and 15% wt.). As previously discussed, the morphology of the electrospun nanostructures largely depends on these variables and also strongly affects the friction behavior of the resulting greases, as shown in Figure 5. The friction coefficient generally decreases when increasing both the concentration of the feeding solution to the electrospinning chamber and the EC proportion in the nanostructures. These results suggest that more homogeneous and fiber-dominated mats are able to more steadily release the oil entrapped in the three-dimensional network, facilitating a regular replenishment in the contact, rather than nanostructures with the prevalence of particles or beads inserted in the fibers where the oil is much more readily separated (see oil separation data in Table S3 for selected samples). In general, the contact replenishment capacity of greases thickened with LSL/EC nanostructures differing in the LSL:EC ratio at the same concentration (20% wt.) seems not to be very different, being the morphological characteristics that govern the tribological performance. In fact, the wear scar volume produced in the steel plates was also significantly reduced when using more homogeneous and fiber-dominated electrospun nanofibrous webs as thickeners (see Figure 6), i.e., when increasing the EC proportion, which produced more viscous greases. On the other hand, the achievement of bead-free nanofiber webs, or, at least, a significant reduction in the number of beads, must also minimize the negative contribution of lignin particles in the tribological contact.

Finally, the friction coefficient values obtained using only castor oil as a lubricant (i.e., the nanofiber-free base oil) are only slightly lower (see Table S2) than those found with fiber-dominated uniform nanostructures (lower values in Figure 5), which supports the idea that friction reduction is mainly driven by a steady oil release from the nanofiber webs.

### 3.5. Influence of the Co-Spinning Polymer on the Tribological Properties of Electrospun Nanostructure-Thickened Lubricating Greases

**Figure 7:**
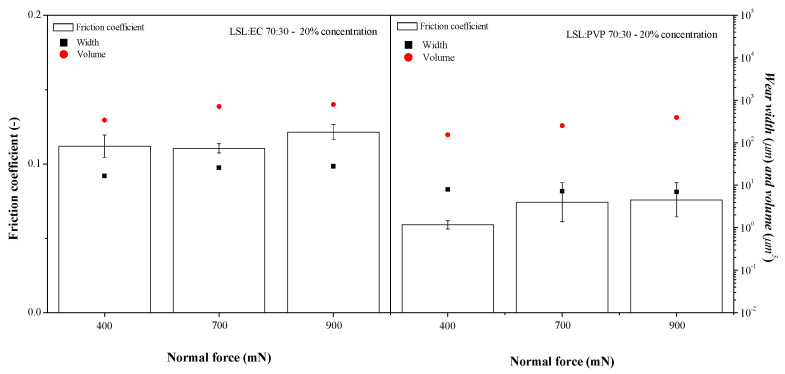
Comparison of the values of the friction coefficient and wear width and volumes obtained with lubricating greases thickened with LSL/EC and LSL/PVP electrospun nanostructures for the same LSLS:co-spinning polymer ratio (70:30 *w*/*w*) and concentration (20%).

As above mentioned, when replacing EC with PVP as a co-spinning polymer, more homogeneous lignin nanofiber webs with a decreased amount of beads were generally obtained. Figure 7 illustrates the impact of replacing the co-spinning polymer on the friction and wear response of the resulting greases thickened with the electrospun nanostructures. As may be seen, the LSL/PVP nanostructures, under the same electrospinning processing conditions (70:30 LSL:co-spinning polymer ratio, 10% feeding solution) and thickener concentration (20% wt.), provide better lubrication performance to the greases in terms of lower friction coefficient values and reduced dimensions of wear grooves. Again, the lowest friction coefficient values are very similar to those obtained with neat castor oil (Table S2). Particularly, lower friction values were measured using greases containing 10% wt. of LSL/PVP-based nanostructures (see Figure S3 in the Supplementary Materials), where again, increasing the amount of the co-spinning polymer, PVP in this case, proved to be beneficial.

### 3.6. Effect of Morphological Parameters on the Friction and Wear Behavior of Electrospun Nanostructure-Thickened Lubricating Greases

Aiming to better elucidate the role of electrospun nanostructure morphology on the tribological response of the resulting greases, some structural parameters such as the average fiber diameter and the nanostructure porosity were calculated from SEM observations and further correlated with the friction coefficient and dimensions of the wear grooves. Although the number of nanoparticles and beads in the electrospun nanostructures is actually an influencing parameter, in these particular lignin webs, it is inversely correlated with fiber diameter, whereas it is proportional to the network porosity. Therefore, electrospun nanostructures with low values of fiber diameters and high porosity are either generally dominated by nanoparticles or composed of BOAS morphologies with a significant number of beads.

**Figure 8:**
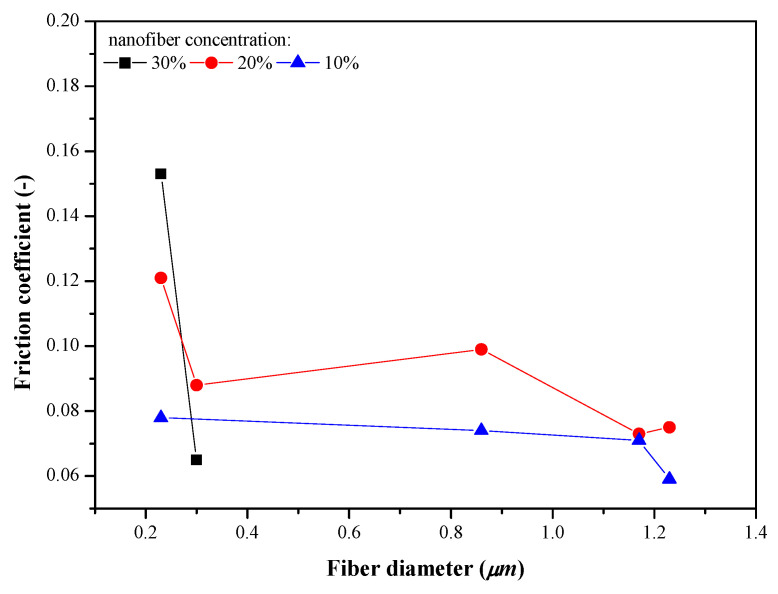
Evolution of the friction coefficient with the nanofiber diameter for greases thickened with LSL/EC electrospun nanofibrous webs at different concentrations (normal force applied: 900 mN).

Figure 8 shows the evolution of the friction coefficient with fiber diameter for greases thickened with LSL/EC nanofibrous webs at the three different concentrations studied. As can be seen, nanostructures with larger average fiber diameters tend to reduce friction in any case. As mentioned, it must be taken into account that smaller fiber diameters are also generally associated with the presence of beads in the nanostructure, whereas larger diameters correspond to more homogeneous and fiber-dominated webs. On the contrary, no significant influence of fiber diameter on wear mark dimensions was evinced.

**Figure 9:**
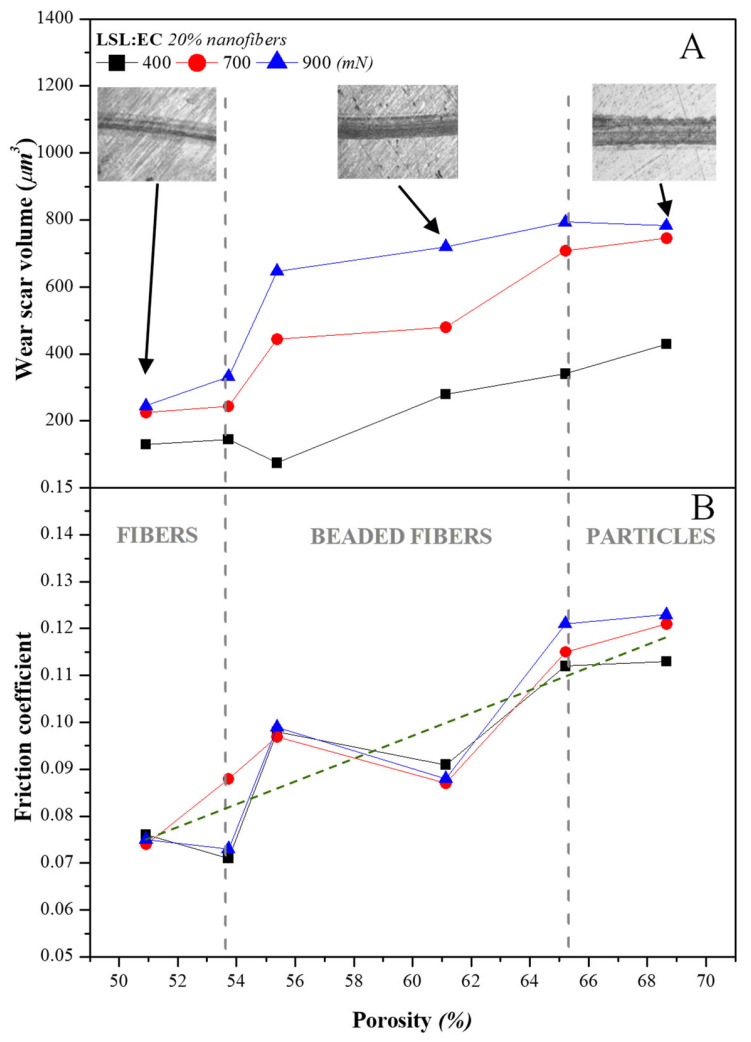
Evolution of (**A**) the wear scar volume and (**B**) the friction coefficient with the porosity of LSL/EC electrospun nanofibrous webs used to thicken greases (20% nanofiber concentration). The green dotted line in (**B**) corresponds to a linear fit y = −0.05 + 0.02x (R^2^ = 0.879), considering all experimental values regardless of the normal load applied.

Even more relevant is the influence of the nanostructure porosity. High porosity is one of the most striking characteristics of the electrospun lignin networks used as thickener agents, which roughly ranges from 50 to 70%, as well as their ability to adsorb or entrap vegetable oils in the voids (see for instance refs. [35,36]). As can be seen in Figure 9, the friction coefficient and the wear scar volume increase with the porosity of the electrospun lignin network. In particular, the friction coefficient correlates linearly with the porosity of the LSL/EC nanofibrous web regardless of the normal force applied in the experimental range analyzed (see linear fit in Figure 9B). In principle, these results may seem unexpected since highly porous networks should facilitate oil release and the replenishment of the lubricated contact. However, it must be taken again into account that, in this case, the more porous electrospun networks correspond to nanostructures dominated by the presence of particles or BOAS. Therefore, two possible and compatible hypotheses must be considered. On the one hand, nanofibers may penetrate into the contact more easily than particles, increasing the film thickness and preventing wear. On the contrary, lignin particles find it more difficult to come into the tribological contact due to their size and are probably accumulated at the inlet zone, particularly under pure sliding conditions, and the bled oil inversely flows under a pressure gradient, causing inlet starvation and more severe wear. On the other hand, although not so porous, a well-developed and homogeneous fibrous network is able to retain but also release oil by the action of an external load more steadily, acting as a sponge and favoring a gradual replenishment of the lubricated contact.

## 4. Conclusions

Electrospun lignin nanostructures obtained using EC or PVP as co-spinning polymers were able to form stable gel-like colloidal dispersions in castor oil at concentrations between 10 and 30% wt. through the formation of percolation networks and can be satisfactorily proposed as cost-effective and renewable thickeners for bio-based lubricating grease formulations.

Friction and wear investigated in a nanotribometer using a steel–steel ball-on-disc configuration was reduced when using lubricating greases thickened with homogeneous bead-free nanofiber mats rather than nanostructures dominated by the presence of particles or beaded fibers. Obtaining homogeneous nanofibrous webs using electrospinning was favored by reducing the lignin:co-spinning polymer ratio or by increasing the concentration of the polymeric feeding solution, yielding larger average fiber diameters and lower porosity values.

The use of PVP as a co-spinning polymer resulted in nanofibrous webs with enhanced friction and wear performance in comparison with ethylcellulose, although ethylcellulose also provided good results at high proportions, and it is preferred in terms of renewability.

In general, according to the experimental results, it may be concluded that well-developed uniform fibrous lignin networks having larger average fiber diameters and lower porosity are able to retain but satisfactorily release the castor oil by the action of the normal load, thus providing a steady replenishment of the lubricated contact.

The lowest friction coefficient values obtained with these well-developed nanofiber webs are very similar to those found with castor oil, which supports the idea that a steady oil release is the main mechanism governing the anti-friction properties of these bio-based greases. Moreover, it was hypothesized that nanofibers are able to better penetrate into the tribological contact much more easily than lignin particles or beaded fibers and thus better prevent wear.

Finally, friction and wear generally increased with thickener concentration, especially at the higher normal loads applied, which must be attributed to a decrease in both the oil release and the contact replenishment capacity as a consequence of the increased viscosity.

## Data Availability

The data presented in this study are available upon request from the corresponding author.

## Supplementary Materials

The following supporting information can be downloaded at: https://www.mdpi.com/article/10.3390/nano13212852/s1. Figure S1: Viscosity vs. shear rate plots for lubricating greases thickened with (a) 70:30 LSL/EC and (b) 70:30 LSL/PVP electrospun nanostructures at different concentrations (10%, 20% and 30% wt.); Figure S2: Viscosity vs. shear rate plots for lubricating greases thickened with LSL/EC electrospun nanostructures differing in the LSL:EC weight ratio (thickener concentration 20% wt.); Table S1: Values of the Sisko model parameters obtained from fitting the viscous flow curves of greases thickened with electrospun nanostructures to Equation (1); Table S2: Friction coefficient values obtained using castor oil as the lubricant; Table S3: Oil separation after centrifugation for selected greases thickened with different electrospun LSL:EC nanostructures; Figure S3: Values of the friction coefficient obtained with lubricating greases thickened with LSL/PVP electrospun nanostructures with different LSL:PVP ratios (10% nanofiber concentration).
