# Supplementary material for: Assessment of the Tribological Performance of Electrospun Lignin Nanofibrous Web-Thickened Bio-Based Greases in a Nanotribometer
Source: Nanomaterials (Basel). 2023 Oct 27;13(21):2852. doi: 10.3390/nano13212852 (PMC10649236; doi:10.3390/nano13212852)
Supplement: Supplementary file 1 [file nanomaterials-13-02852-s001.zip › nanomaterials-2657696-supplementary.pdf]

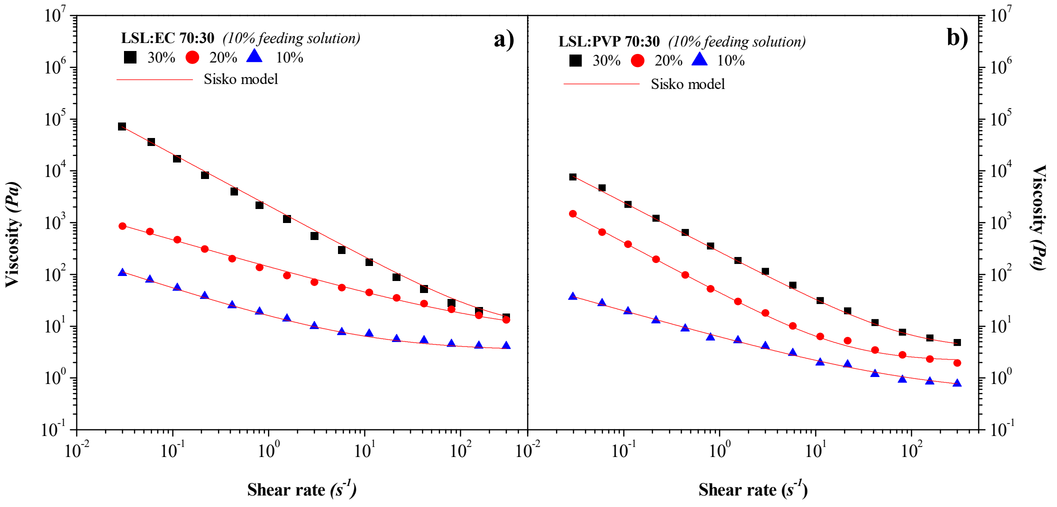


**Figure S1**. Viscosity vs. shear rate plots for lubricating greases thickened with a) 70:30 LSL/EC and b) 70:30 LSL/PVP electrospun nanostructures, at different concentrations (10%, 20% and 30% wt).

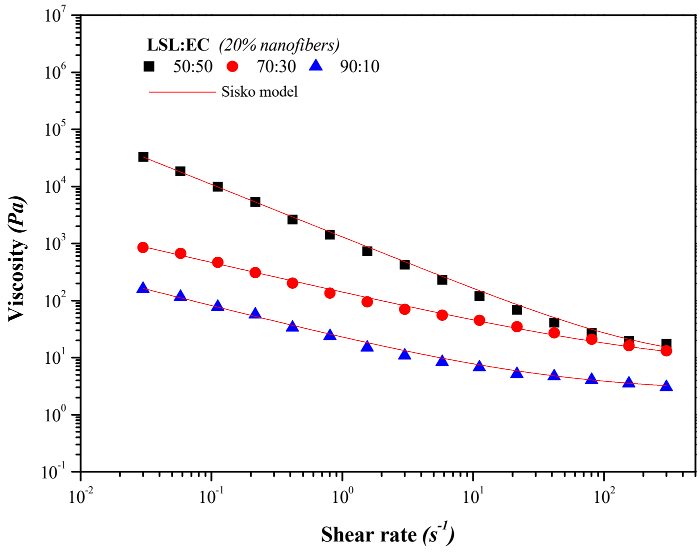


**Figure S2.** Viscosity vs. shear rate plots for lubricating greases thickened with LSL/EC electrospun nanostructures differing in the LSL:EC weight ratio (thickener concentration 20% wt).

**Table S1**. Values of the Sisko model parameters obtained from the fitting of the viscous flow curves of greases thickened with electrospun nanostructures to eq. (1).

| LSL:co-spinning polymer ratio (w/w) | Thickener concentration (wt.%) | $K$ (Pa s$^n$) | $n$ (-) | $\eta_\infty$ (Pa·s) |
|---|---|---|---|---|
| **50:50 LSL:EC** | 10 | 119.1 | 0.31 | 4.7 |
| | 20 | 1297.4 | 0.08 | 8.5 |
| **70:30 LSL:EC** | 10 | 12.7 | 0.43 | 3.3 |
| | 20 | 134.5 | 0.49 | 6.4 |
| | 30 | 2111.7 | 0.001 | 8.4 |
| **90:10 LSL:EC** | 20 | 20.5 | 0.40 | 2.5 |
| | 30 | 59.1 | 0.59 | 3.9 |
| **50:50 LSL:PVP** | 10 | 9.4 | 0.32 | 1.2 |
| **70:30 LSL:PVP** | 10 | 5.75 | 0.47 | 0.5 |
| | 20 | 42.4 | 0.01 | 2.1 |
| | 30 | 268.5 | 0.04 | 3.5 |
| **90:10 LSL:PVP** | 30 | 5.69 | 0.47 | 3.9 |

**Table S2.** Friction coefficient values obtained using castor oil as lubricant.

| Normal force (mN) | Friction coefficient (-) |
|---|---|
| 400 | 0.061±0.004 |
| 700 | 0.055±0.002 |
| 900 | 0.063±0.001 |

**Table S3.** Oil separation after centrifugation for selected greases thickened with different electrospun LSL:EC nanostructures (obtained from 10% feeding solutions).

| LSL:EC ratio (w/w) | Thickener concentration (wt.%) | Oil separation (wt. %) |
|---|---|---|
| **50:50** | 10 | 4 |
| | 20 | 0 |
| **70:30** | 10 | 43 |
| | 20 | 5 |
| | 30 | 0 |
| **90:10** | 20 | 70 |
| | 30 | 24 |

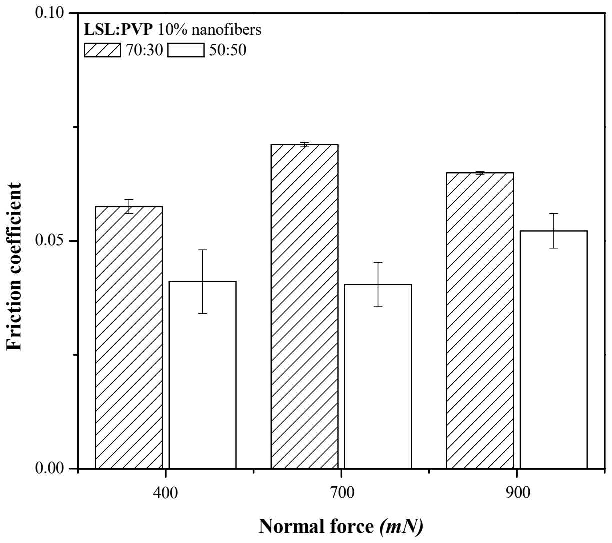


**Figure S3.** Values of the friction coefficient obtained with lubricating greases thickened with LSL/PVP electrospun nanostructures with different LSL:PVP ratios (10% nanofiber concentration).
